# A systematic review of chatbot-assisted interventions for substance use

**DOI:** 10.3389/fpsyt.2024.1456689

**Published:** 2024-09-10

**Authors:** Serim Lee, Jiyoung Yoon, Yeonjee Cho, JongSerl Chun

**Affiliations:** ^1^ Department of Social Welfare, Ewha Womans University, Seoul, Republic of Korea; ^2^ School of Public Health, University at Albany, State University of New York, Rensselaer, NY, United States

**Keywords:** chatbot, artificial intelligence, substance use, intervention, systematic review

## Abstract

**Objectives:**

This study systematically reviewed research on the utilization of chatbot-related technologies for the prevention, assessment, and treatment of various substance uses, including alcohol, nicotine, and other drugs.

**Methods:**

Following PRISMA guidelines, 28 articles were selected for final analysis from an initial screening of 998 references. Data were coded for multiple components, including study characteristics, intervention types, intervention contents, sample characteristics, substance use details, measurement tools, and main findings, particularly emphasizing the effectiveness of chatbot-assisted interventions on substance use and the facilitators and barriers affecting program effectiveness.

**Results:**

Half of the studies specifically targeted smoking. Furthermore, over 85% of interventions were designed to treat substance use, with 7.14% focusing on prevention and 3.57% on assessment. Perceptions of effectiveness in quitting substance use varied, ranging from 25% to 50%, while for reduced substance use, percentages ranged from 66.67% to 83.33%. Among the studies assessing statistical effectiveness (46.43%), all experimental studies, including quasi-experiments, demonstrated significant and valid effects. Notably, 30% of studies emphasized personalization and providing relevant tips or information as key facilitators.

**Conclusion:**

This study offers valuable insights into the development and validation of chatbot-assisted interventions, thereby establishing a robust foundation for their efficacy.

## 1 Introduction

Chatbots, based on human-computer interaction systems (1, 2), utilize either rule-based systems, which rely on rules defined by expert knowledge (e.g., decision trees), or natural language processing, a branch of artificial intelligence (AI), to emulate a real-time conversation (3). Modern chatbots use a combination of these two approaches (3).

With the development of AI, chatbots are being utilized across diverse sectors such as education, health, entertainment, and business, including e-commerce (2), employing spoken, written, and visual languages (4). In the health care sector, chatbots have been used to educate, prevent, support, treat, and diagnose people with diverse medical needs, including addiction (5–7). Chatbots offer intelligent guidance, enhance productivity through automated engagement, provide on-demand accessibility, mitigate user judgment, and exhibit enduring patience for clients (2, 5, 8).

These characteristics have underscored the utility of emerging technologies like chatbots as a telehealth solution for various mental health challenges, which have become more prevalent amidst the constraints on in-person services since the COVID-19 pandemic (9, 10). Particularly noteworthy is the capacity of chatbot technology to offer emotional support to users in an interactive and empathetic manner, making it appealing for mental health interventions by facilitating the formation of therapeutic relationships (9). Previous studies have provided evidence for the feasibility of utilizing these digital tools to foster “digital therapeutic alliances” (9, 11). Research indicates that some chatbot users find comfort in anonymous interactions, providing a platform for intervention for those averse to traditional counseling settings (9, 12). Furthermore, interventions assisted by chatbots, accessible through smartphones, laptops, and tablets, offer several advantages for addiction management and treatment by providing immediate support without the stigma often associated with seeking help within the community (10).

Individuals grappling with substance use disorders are especially vulnerable to intense negative emotions like guilt, shame, or embarrassment when contemplating seeking help, posing a substantial hurdle to treatment initiation (5, 13). However, interventions facilitated by chatbots can mitigate these obstacles owing to their anonymous and non-face-to-face accessibility (14). Additionally, their capacity for individualized, round-the-clock support without succumbing to fatigue or burnout, even amidst recurring relapses driven by urges and cravings characteristic of addiction (15, 16), positions chatbots as a significant advancement beyond conventional mobile health technologies such as text or instant messaging (14, 17). Chatbot-assist interventions can provide support similar to human interaction and offer customized assistance tailored to individual recovery levels or prevention needs (10).

Hence, within the domain of substance use, encompassing alcohol, smoking, and drugs, an expanding body of literature validates the efficacy of chatbot-assisted approaches for assessment, prevention, and treatment methods (18–20). As a result, systematic reviews have been conducted to identify the effectiveness and research trends of chatbot-based intervention studies for substance use disorders. However, these studies have been limited by their broad scope, which includes not only substance use disorders but also mental health (17) or by excluding nicotine from the category of addictive substances (5). In particular, Ogilvie et al.’s study (5) underscores the uncertain effectiveness of chatbot-assisted intervention for substance use based on a review of only six studies. However, contrasting findings emerge from a scoping review focusing on chatbots for smoking cessation, which predominantly suggests their effectiveness (21). In summary, a more comprehensive investigation is needed, one that encompasses substance use and rigorously compares effectiveness across different types of substances.

This study aims to address this gap by conducting a thorough systematic review, examining the utilization of chatbot-related technologies for prevention, assessment, and treatment across all substance use types, including alcohol, nicotine, and other drugs. We specifically focus our review on digital mental health interventions that encompass diagnosis or screening, symptom management and behavior change, prevention, or therapeutic content delivery (22).

## 2 Methods

### 2.1 Search strategy

The systematic review meticulously analyzed records from four databases—PubMed, PsycINFO, Scopus, and CINAHL—up to March 7, 2024, marking the start of the present study. We did not specify a start date for the article inclusion criteria, meaning that all articles, regardless of their publication date, were included from the time the first related article appeared until March 7, 2024. We chose these databases due to their widespread use in systematic reviews covering similar research topics (23). We utilized two sets of distinct topic keywords: 1) chatbot, conversational agent, and conversational artificial intelligence; and 2) substance use, alcohol, smoking, and drug.

### 2.2 Study selection

Following the PRISMA guideline, the present study progressed through distinct stages—identification, screening (including eligibility assessment), and inclusion (24)—to compile relevant sources. All 998 references from each database were imported into the Covidence program (25), which automatically removed 129 duplicates, leaving 869 records for subsequent title and abstract screening. Three out of four reviewers searched the databases using keywords and imported the results into the Covidence program, with oversight from the fourth reviewer.

**Figure 1:**
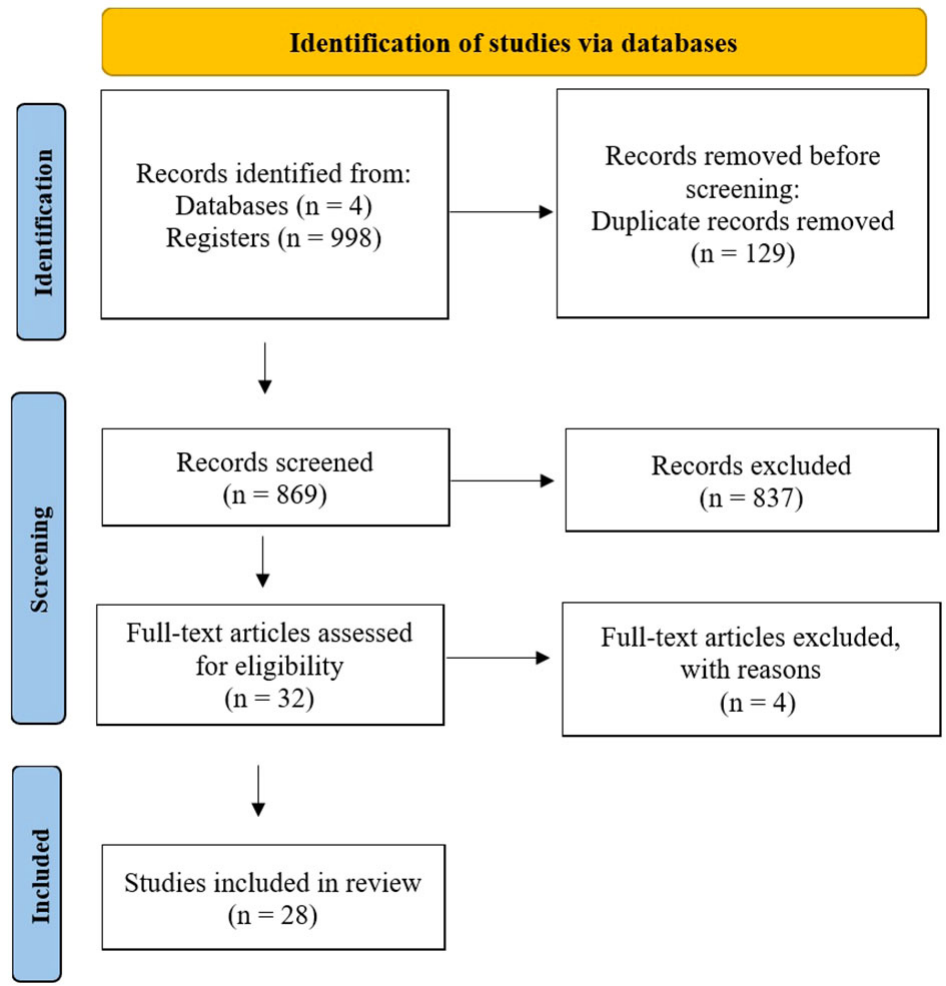
PRISMA Flow Chart.

The systematic review encompassed studies meeting specific inclusion and exclusion criteria. Inclusion criteria required studies to 1) be peer-reviewed articles published in English regardless of the country where the studies were conducted, 2) contain information on any type of chatbot-assisted intervention (voice, internet, and messenger platform) for substance use, 3) include experimental, non-experimental, and qualitative studies, 4) provide all necessary data information (e.g., sample size, odds ratio, 95% CI, or other effect size values), and 5) be rated as “fair” or “good” based on the National Institute of Health (NIH) quality assessment tool (26). Conversely, exclusion criteria encompass studies that are 1) master’s theses or doctoral dissertations, 2) commentary and editorials, and 3) review papers, including systematic reviews and meta-analyses. Three out of four reviewers independently rated each article as “yes,” “no,” or “maybe” based on the criteria. In cases of conflicting ratings, the reviewers discussed them together to reach a consensus, with oversight from the fourth reviewer. From the first screening stage, 837 irrelevant records were removed, resulting in 32 articles advancing to full-text review. Four articles were excluded based on these criteria, leaving 28 articles for final analysis (See **Figure 1**).

### 2.3 Data extraction and analysis

Prior to the coding process, approximately 10% of the final sample was randomly selected by the authors for double screening to ensure consistency among raters (27, 28). Three reviewers conducted individual rating and coding of articles in the Excel spreadsheet matrix. The authors collectively discussed and resolved any differences in wording choice. The coding encompassed various details, including author and year, study type, data source, sampling methods, sample characteristics (e.g., size, age range, mean age, gender distribution, racial demographics), type of chatbot-assisted intervention (e.g., assessment, prevention, treatment), contents of the intervention (e.g., theoretical framework, duration, session), type of substance use, measurement tools for substance use, and main findings/outcomes, which include the effectiveness of chatbot-assisted interventions on substance use and the facilitators and barriers impacting their effectiveness.

## 3 Results

### 3.1 Study characteristics (date of publication, study type, data source, and research methods)

**Figure 2:**
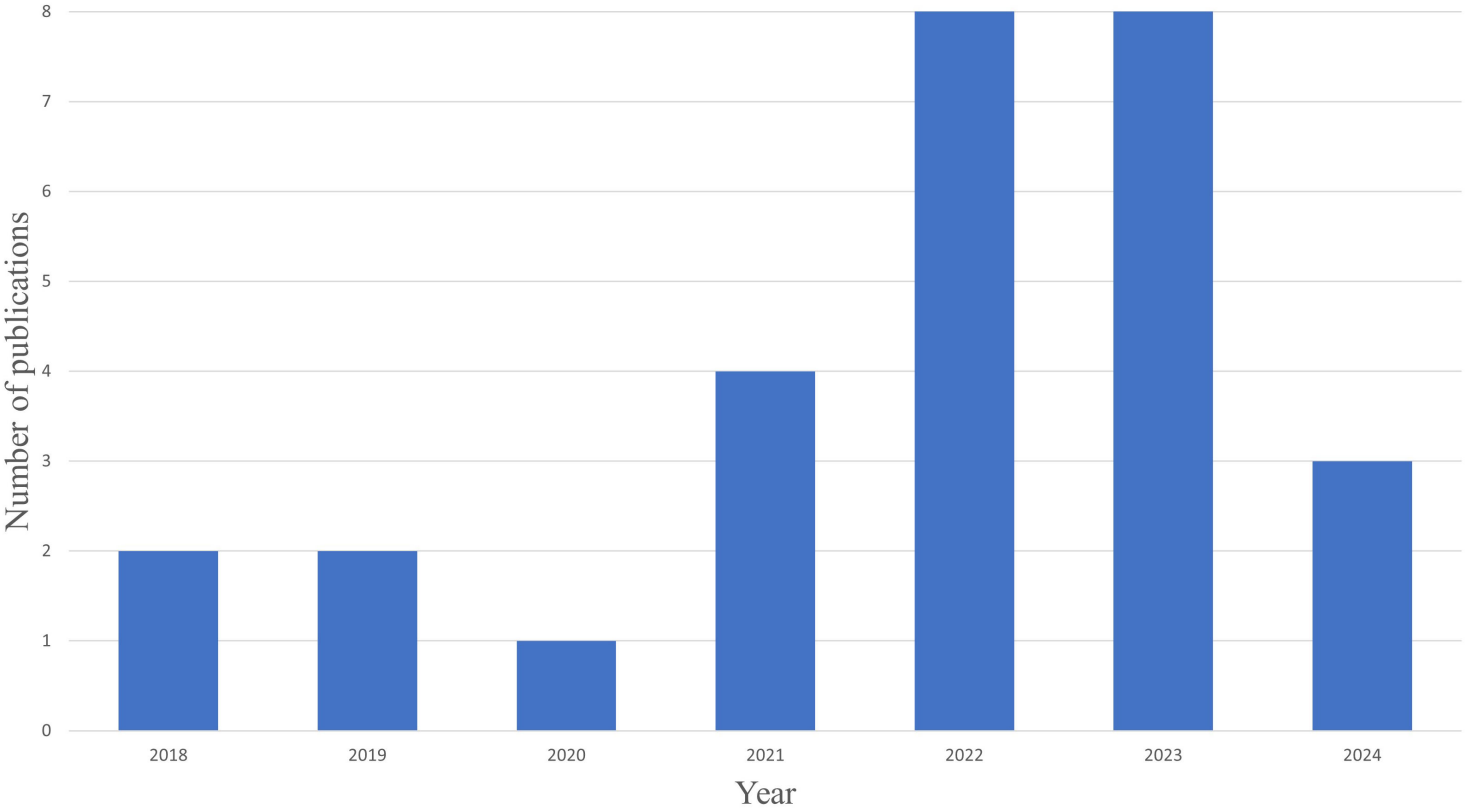
Date of Publication.

A total of 28 studies met our inclusion criteria. All studies included in this analysis were conducted between 2018 and 2024. Most studies (57.14%; 16 out of 28) were published in 2022 and 2023 (See **Figure 2**).

Reviewed studies collected primary data from diverse channels, with 64.29% (18 out of 28) using online platforms (such as web-based platforms, social media, and telephone) and 35.71% (10 out of 28) using offline sources, encompassing open advertisement, clinical, community-based settings, and school.

In our analysis of 28 studies, we identified three primary study types. Quantitative studies constituted 60.71% (*n* = 17), followed by mixed methods studies at 28.57% (*n* = 8), and qualitative studies at 10.71% (*n* = 3). Most (52.94%, 9 out of 17) quantitative studies employed experimental designs, whereas 35.29% (6 out of 17) utilized quasi-experimental designs, and 11.76% (2 out of 17) adopted non-experimental designs. Various statistical analyses (e.g., frequency analysis, t-test, correlation, logistic regression model, generalized linear mixed model, chi-square test, ANOVA, etc.) were conducted across the studies, with the t-test being the most frequently utilized method (32.14%, 9 out of 28). This choice was primarily motivated by the need to discern differences between groups within the dataset. In mixed methods studies, researchers employed a range of data collection methods, such as interviews (29), surveys (30–33), transcript analysis (3), literature reviews (30), and observations (34). The most common combination entailed open-ended questions for qualitative data and rating on a scale such as a Likert scale for quantitative data (37.5%, 3 out of 8). The qualitative data were gathered through semi-structured telephone interviews (19, 35), in-depth, one-on-one semi-structured interviews (36), and focus group discussions (19). Thematic analysis is applied to all three qualitative studies, which aim to obtain users’ experiences, feedback, and opinions.

In this study, we categorized the study stage into two distinct stages: planning and testing of the chatbot program. The planning stage, which encompasses research protocol, accounted for 25% (7 out of 28) of the studies. This stage involved protocol studies (29, 31, 37, 38) and design and development, such as a methodological framework for the emulation of human-conversational agent interactions that build on social media sequencing (39). The testing stage, comprising 75% (21 out of 28) of the studies, was conducted to investigate the feasibility and preliminary efficacy outcomes of chatbot interventions. Specifically, nine out of the 28 studies (32.14%) analyzed program effectiveness through descriptive analysis (frequency), three (10.71%) examined program effectiveness through descriptive analysis (mean), while 14 (50%) assessed the effectiveness of chatbot-based interventions for substance use through experimental and quasi-experimental designs (not exclusively).

### 3.2 Types and contents of chatbot-assisted interventions

Of the 28 papers reviewed, 18 (64.29%) present theories or therapies that form the basis of chatbot program content. Among these 18, the most frequent approach (9 papers, 50%) was the fusion of various theories, such as dialectical behavior therapy, mindfulness, problem-solving, and person-centered therapy, primarily based on cognitive behavioral therapy and motivational interviewing (MI). Next, three studies (16.66%) applied only MI, and another three studies (16.66%) presented evidence based on the World Health Organization (WHO) or the country’s standardized intervention manual. Acceptance and commitment therapy (5.55%), mindfulness-based relapse prevention (5.55%), and behavioral theory (5.55%) were each confirmed in one study, respectively.

A total of 22 studies (78.57%) presented specific program content. The content varied substantially depending on the underlying theory or therapy and the intervention period. Programs often included motivation-boosting messages or feedback (40, 41), psychoeducation, and emotion management related to craving and stress (29, 42). Additionally, daily notifications, craving tracking, goal setting for substance use cessation, and daily feedback or guidance were provided (29, 35). Six articles (27.27%) provided session-type content, which organizes content sequentially as users access it. Three articles (13.63%) provided module-type content, which bundles content by specific topics, allowing users to select topics based on their interests. The remaining 13 articles (59.09%) did not disclose specific methods. The number of sessions ranged from 1 to 14, while the number of modules ranged from 6 to 8.

Among the chatbot-assisted programs, 24 out of 28 (85.72%) were designed to treat substance use by changing the user’s behavior or cognition, followed by two programs (7.14%) focused on prevention and one (3.57%) on assessment. Sixteen out of 28 studies (57.14%) reported the intervention period of the chatbot programs. The intervention periods varied widely, ranging from a single session to a maximum of six months. The most common duration was an 8-week intervention, reported in 4 out of 16 studies (25.0%), followed by 2-week interventions (18.75%), 10-week interventions (12.50%), 12-week interventions (12.50%), and 6-month interventions (12.50%), with each of these durations reported in two studies. Additionally, one study each reported interventions lasting 1 day (6.25%), 16 weeks (6.25%), and 14 weeks (6.25%).

### 3.3 Sample characteristics (sampling method)

Among the 28 studies reviewed, 23 (82.14%) involved sampling human participants. Only three studies (10.71%) explicitly stated the sampling methods used, encompassing purposive sampling (3, 35) and convenience sampling (43). In contrast, the remaining studies briefly described the recruitment process, utilizing web-based platforms, social media, Facebook, hospitals, clinical and community-based settings, flyers, universities, and psychiatric centers, without specifying the sampling methods employed. The mean sample size across the studies was 2,739 (Standard deviation; *SD* = 11,618.34), with a considerable range from 6 (44) to 57,214 participants (45). Of the 23 studies, 15 (21.74%) reported the mean age of participants, with an average of 36.76 (*SD* = 10.35), ranging from 15 to 76 years old.

Regarding gender representation, 18 out of 28 studies (64.29%) disclosed the percentage of male and female participants included in their studies. On average, the percentage of male participants was 42.62% (*SD* = 20.17), while the percentage of female participants was 45.06% (*SD* = 21.07). The mean percentage of participants identifying as other genders was 3.03% (*SD* = 2.11).

Additionally, 7 out of 28 studies (25%) reported participants’ race/ethnicity. On average, the percentage of White, Black, Hispanic, Asian, and other participants was 66.87% (*SD* = 15.73), 17.67% (*SD* = 15.87), 23.78% (*SD* = 35.12), 5.14% (*SD* = 1.36), and 9.62% (*SD* = 9.29), respectively.

Three out of the 28 studies (10.71%) employed text sampling methods, which included the following: a “sample of recorded telephone-counseling sessions” focusing on various aspects of smoking cessation (3), “QuitNet Peer Interactions” comprising 2.23 million labeled peer interactions with 2,005 manually annotated messages (39), and an analysis of “236,000 sessions in Pahola’s page” accessed by 188,000 users (34).

### 3.4 Target and measurement tools used to assess

Out of 28 studies, 50% (*n* = 14) focused specifically on smoking (i.e., tobacco, nicotine), while 21.43% (*n* = 6) adopted a comprehensive approach to substance use that included alcohol, tobacco, cannabis, methamphetamine, cocaine, and pharmaceutical medications. Furthermore, 17.86% (*n* = 5) of the studies focused on alcohol use, 7.14% (*n* = 2) targeted methamphetamine use, and 3.57% (*n* = 1) addressed both alcohol and tobacco concurrently.

Out of 28 studies, 16 (57.14%) reported measurement tools for substance use. Nine out of 16 studies (56.25%) utilized standardized measurement tools to measure substance use, such as the Heaviness of Smoking Index (40), CAGE Adapted to Include Drugs, Drug Abuse Screening Test (DAST-10), Brief Situational Confidence Questionnaire (38, 42, 46), Short Inventory of Problems—Alcohol and Drugs (38, 42), Alcohol Use Disorders Identification Test (AUDIT-C) (46), US AUDIT, Readiness to Change Questionnaire, Short Inventory of Problems – Revised, and Timeline Followback (47), Cigarette Dependence Scale-5 (CDS-5), CAGE (48), Fagerstrom Test of Nicotine Dependence (FTND) and Smoking Abstinence Self-Efficacy Questionnaire (31), FTND (49), Stages of Change Readiness and Treatment Eagerness Scale, and Visual Analogue Scale (50).

Three studies (18.75%) used medical tests, including the Drug Urine Test in conjunction with DSM-5 criteria (10) and the Co-oximetry Test, which measured exhaled air in parts per million (37, 51). Three studies (18.75%) solely relied on non-standardized tools such as “smoke at least 1 cigarette daily” (44), “time to first cigarette,” “cigarettes per day” (45), “at risk-drinking in the preceding 30 days,” “total number of alcoholic drinks consumed in the preceding 30 days,” “tobacco/e-cigarette smoking, preceding 30 days,” “quantity of cigarettes smoked preceding 30 days,” “cannabis use, preceding 30 days,” “cannabis use days, preceding 30 days” (43). One study (6.25%) (52) utilized both standardized (Drinking Refusal Self-Efficacy Questionnaire) and non-standardized measurement tools (“binge drinking in past 30 days,” “maximum number of alcoholic standard drinks consumed in past 30 days,” “total number of alcoholic standard drinks consumed in past 30 days”).

### 3.5 Main findings

#### 3.5.1 Effectiveness of program – Descriptive analysis

Nine out of the 28 studies (32.14%) analyzed program effectiveness through descriptive analysis (frequency), categorizing responses into seven themes: 1) Helpful for substance use, 2) Quit/cut substance use, 3) Reduced/cut down substance use, 4) Positive feelings, 5) Willingness to recommend or participate again, 6) Easiness/comprehensibility, and 7) Lifelike/related to their situation.

The percentage of respondents indicating programs as 1) Helpful for substance use varied from 8.3% (smoking) (53) to 84.6% (alcohol) (52), 85% (methamphetamine) (10), and 100% (smoking) (44). The percentage of respondents indicating they 2) Quit/cut substance use ranged from 25%-40% (attempt to quit) (40), 33.33% (quit smoking) (36), 50% (setting a quit smoking date within 14 days) (44), to 50% (choosing to cut back on drinking; 75% of Spanish, 60% of English users, and 50% of Portuguese users) (34). The percentages of respondents who 3) Reduced/cut down substance use were 66.67% (cut down smoking) (36), 70.5% (made some kind of smoking reduction attempt) (40), and 83.33% (reduced smoking) (44). Regarding the measurement of sustained time for stopping/reducing substance use, 12 out of 28 studies (42.9%) reported the duration measured. The most common period was one month (*n* = 5), followed by six months (*n* = 3), one year (*n* = 2), one week (*n* = 1), and two weeks (*n* = 1).

Regarding 4) Positive feelings, “rate positively” ranged from 94% (46) to 96% (42). “Pleasant” was reported at 34.7% (53), “enjoyed” at 87.9% (52), “impressive” at 100% (44), and “feeling cared” at 67% (10). “Satisfaction” ranged from 84% (10) to 100% (44). Regarding 5) Willingness to recommend or participate again, “would Recommend” ranged from 67% (10) to 76.2% (52) and 86% (42). Additionally, 89.1% answered that they would participate again (52). For 6) Easiness/comprehensibility, the rate of easy interaction was reported at 83.3% (44), and the rate of comprehensibility was 100.0% (52). Finally, regarding 7) Lifelike/related to their situation, 70.8% indicated they felt it was relevant to their individual situations (52), and 66.67% felt it was lifelike (44).

Three out of the 28 studies (10.71%) examined program effectiveness through descriptive analysis (mean). Boustani et al. (33) found that participants reported high acceptability and utility of the technology (Mean (M); *M* = 2.31, *SD* = 1.05, out of 7), high engagement (*M*= 2.86, *SD* = 0.96, out of 7), and a high number of human-like traits (*M* = 2.07, *SD* = 0.89, out of 7) of a chatbot-based intervention for alcohol. Auriacombe et al. (48) also reported high Acceptability E-Scale scores (24.8; out of 30, *SD* = 4.2) of a chatbot-based intervention for alcohol and tobacco use. Loveys et al. (32) revealed that users reported a positive overall experience with a chatbot-based intervention for tobacco use (*M* = 3.17, *SD* = 0.82, out of 4) and found the chatbot to provide useful information and advice (*M* = 3.21, *SD* = 0.92, out of 4).

#### 3.5.2 Program effectiveness—Experimental and quasi-experimental designs

In 13 out of 28 studies (46.43%), the effectiveness of chatbot-based interventions for substance use was examined through experimental and quasi-experimental designs. Among these 13 studies, 100% reported significant effectiveness. For smoking, intention to quit (*M* change 0.8, standard error (*SE*); *SE* = 0.1, *p* <.001, respectively) (49), motivation to quit (*F* (1,151) = 32.67, *p* < .001) (41), quit success (79.55% in the intervention group vs. 73.35% in the control group, *OR* for the adjusted model; *OR_adj_* = 1.36, 95% confidence interval (*CI*); *CI* = 1.16-1.61, *p* < .001) (45), quitting confidence (*M* change 0.1, *SD* = 2.0-2.3, *p* <.001), quitting importance (*M* change = 0.7, *SD* = 2.0, *p* < .001), and quitting readiness (*M* change 0.4, *SD* = 1.7, *p* <.01) (40); biochemically validated abstinence rate of smoking (26% for the intervention group vs. 18.8% in the control group, odds ratio (*OR*); *OR* = 1.52, 95% *CI* = 1.00-2.31, *p* = .05) (20) increased after exposure compared to baseline or were higher in the intervention group compared to the control group. In addition, significant group effects were observed for the 30-day point prevalence for tobacco/e-cigarette smoking (*OR* for the intervention group; *OR_ITT_* = 0.74, 95% *CI* = -0.55-1.01, *OR* for the control group; *OR_CC_* = 0.62, 95% *CI* = 0.40-0.96) (43).

For alcohol, binge drinking (*OR* = 0.32, 95% *CI* = 0.18-0.57, *p* <.001), maximum alcohol consumption (incidence rate ratio (*IRR*); *IRR* = 0.75, 95% *CI* = 0.68-0.82, *p* <.01), and number of standard drinks per month (*IRR* = 0.62, 95% *CI* = 0.58-0.67, *p* < . 01) significantly decreased, while drinking refusal self-efficacy significantly increased (*β* = 0.24, 95% *CI* = 0.06-0.42, *p* = .01) (52). Use of any interventions (chatbot or non-bot app) was shown to predict reduced drinking (*β*= 0.25, 95% *CI* = 0.00-0.01, *p* = .04) (47). Scores on the AUDIT-C (*M* change -1.3, *SD* = 2.6, *p* <.001) significantly decreased (46). Significant group effects were observed for at-risk drinking in the past 30 days (*Cohen’s d* for the intervention group; *Cohen’s d_ITT_* = 0.68, 95% *CI* = 0.52-0.89, *Cohen’s d* for the control group; *Cohen’s d_CC_* = 0.61, 95% *CI* = 0.43-0.84), and total number of alcoholic drinks consumed in the past 30 days (*Cohen’s d_ITT_* = 0.07, *Cohen’s d_CC_* = 0.11) (43).

For methamphetamine, the experimental group had fewer methamphetamine-positive urine samples than the control group (19.5% in the experimental group vs. 29.6% in the control group, *F* = 9.116, *p* = .003) (10). For substance or drug use, treatment motivation for substance use (*p* < .001, *Cohen’s d* = -0.60) (50), motivation for abstaining from drugs (*p* = .045, *Cohen’s d* = -0.30) (50), confidence (*p* < .01, *Cohen’s d* = -0.45) (46, 50), and importance (*p* < .001, *Cohen’s d* = -0.50) (50) significantly increased, while craving (*p* = .01, *Cohen’s d* = 0.038 in Chen et al.’s (50) study and *M* change -0.38, *B*(*SE*) = −.38(0.16), *OR* = 0.69, 95% *CI* = 0.50-0.90 in Prochaska et al.’s (46) study, past-month substance use occasions (*M* change -9.1, *SE* = 2.0 in intervention group vs. *M* change = -3.3, *SE* = 1.8 in control group; *p* = .039, *Eta* ^2^ = .029 in Prochaska et al.’s (42) study and *M* change -9.3, *SD* = 14.1, *p* <.001 in Prochaska et al.’s (46) study), scores on the DAST-10 (*M* change -1.2, *SD* = 2.0, *p* <.001) (46), number of cannabis use days in the past month (*Cohen’s d_ITT_* = 0.06, *Cohen’s d_CC_* = 0.14) (43) significantly decreased.

One study (48) examined chatbot-based assessment for tobacco or alcohol use disorder and found that the chatbot named Embodied Conversational Agent (ECA) was acceptable and valid to screen tobacco or alcohol use disorder among patients not requesting treatment for addiction, as the correlation between the ECA, CDS-5, and CAGE interviews and the paper version questionnaires scores were high [*r*(139) = .944, *p* < .0001 for CDS-5 and *r*(139) = .893, *p* < .0001 for CAGE] (48).

#### 3.5.3 Facilitator or barriers affecting program effectiveness

Ten out of the 28 studies (35.71%) reported facilitators influencing the effectiveness of chatbot interventions. Among these, three (30%) (3, 35, 43) identified personalization (e.g., individualized, personal agency, personalized, etc.) as a key facilitator, while three (30%) (35, 41, 44) emphasized the importance of providing relevant tips or information. Additionally, factors such as younger age, lower severity of substance use (40), reinforcement and positive feedback, friendly and knowledgeable interactions, repetition of key messages, supportive interpersonal relationships (44), immediate access to responses (10), and the perception of conversing with a human (47) were mentioned as facilitators. Moreover, Chen et al. (50) found that patients’ scores on the Generalized Anxiety Disorder-7 assessment (*b* = 3.57, *p* <.001, 95% *CI* 0.80-2.89) and Barratt Impulsiveness Scale-Motor Impulsiveness (*b* = -2.10, *p* = .04, 95% *CI* = -0.094-0.02) were predictors of changes in treatment motivation during treatment.

Six out of the 28 studies (21.43%) reported barriers affecting program effectiveness, including technical problems (e.g., login difficulties, heavy tablets, technical errors) (44, 47), short session durations (41, 44), inappropriate responses (e.g., inappropriate reflections in conversation, repetitiveness of bot conversations, excessive pressure to set a quit date, poor response sequencing, lack of liveliness compared to human interaction) (35, 40, 44), lack of personalization (e.g., receipt of non-tailored daily tips) (35), higher severity of substance use (10), low readiness to change (10), and text-centric chatbots that are perceived as simpler and less engaging compared to those incorporating visual graphs and pictures (47).

#### 3.5.4 Qualitative results

Of the 11 studies employing qualitative methods (eight mixed methods and three qualitative), eight studies presented qualitative results (72.72%). Among these, five (62.5%) utilized a mixed research design, while three (37.5%) employed a purely qualitative research design. The purely qualitative studies included those aimed at identifying users’ needs for program development (3, 30, 39) and assessing usability through experiences with chatbot program users. This variable was investigated via qualitative interviews or open-ended surveys (32, 33, 35, 36).

Research on users’ needs for program development emphasized the presence of individual differences in the situations and characteristics in which substance users feel cravings, highlighting the necessity for chatbot responses to consider this context (39). Studies on the user experience of chatbot programs revealed that users appreciated friendliness and showed interest in interacting with chatbots that had more human-like features (voice, appearance, communication), reporting sufficient acceptability (32, 33, 36). Additionally, users positively evaluated personalized interventions, improved insight into addiction, appropriate ventilation for cravings, and daily tips (35, 36). However, some studies indicated that while chatbot-assisted interventions can provide efficient care, they have limitations in achieving deep, open, empathetic communication, as reported through interviews with users and field counselors (19).

## 4 Discussion

This study aimed to identify and summarize gaps in the published literature on chatbot-assisted interventions for substance use through a systematic review. Half of the studies reviewed specifically targeted smoking, while 21.43% took a comprehensive approach covering various substances; additionally, 17.86% focused solely on alcohol, 7.14% on methamphetamine use, and 3.57% addressed both alcohol and tobacco simultaneously. The fact that most studies focus only on smoking suggests the necessity for future studies to encompass a broader range of substances. In addition, over 85% of chatbot-assisted programs were designed for therapeutic purposes, highlighting the need for the development and validation of more assessment and prevention programs as well. The percentage of respondents reporting chatbot-assisted interventions as helpful for substance use varied widely, ranging from 8.3% to 100%. Similarly, perceptions of effectiveness in quitting substance use ranged from 25% to 50% and from 66.67% to 83.33% for reducing substance use.

Furthermore, a minority of the studies assessed the statistical effectiveness of chatbot-based interventions for substance use using experimental and quasi-experimental designs, emphasizing the need for future research to actively confirm the statistical effectiveness of evidence-based interventions for clients. Among the 46.43% (*n* =13) of studies that assessed statistical effectiveness, all (100%) studies demonstrated significant and valid effects. Focusing specifically on smoking cessation, the interventions led to heightened intention to quit, motivation, success rates, confidence, importance, and readiness to quit among smokers, with post-exposure biochemically validated abstinence rates significantly higher compared to baseline or control groups. Alcohol-related interventions resulted in significant reductions in binge drinking, maximum alcohol consumption, AUDIT-C scores, and monthly standard drink consumption, alongside a noteworthy increase in drinking refusal self-efficacy. For methamphetamine, the experimental group had fewer methamphetamine-positive urine samples than the control group. In the context of substance or drug use, significant increases were found in treatment motivation for substance use, motivation for abstaining from drugs, confidence, and perceived importance, alongside notable decreases in craving, past-month substance use occasions, DAST-10 scores, and the number of cannabis use days in the past month.

All experimental and quasi-experimental studies confirmed that chatbot-assisted programs are effective in promoting awareness and behavior change among substance users. This suggests that chatbot-assisted programs facilitate the delivery of relevant information by providing interventions in an internet environment without physical barriers such as geography and time. Furthermore, the results suggest that frequent exposure and stimulation can be effective. While the theories underlying the content provided by each chatbot program varied, all showed significant effects. That is, some studies compared the effectiveness of chatbots with and without reflection feedback (40) or tested differences based on applied MI and confrontational counseling (49), but these studies found no differences between groups, suggesting that chatbot-based interventions for substance users should focus on stimulating users to inquire about their substance use, engage in feedback conversations, and provide appropriate information daily rather than adhering to specific theories or therapies.

In 35.71% and 21.43% of the studies, facilitators and barriers affecting the effectiveness of chatbot-assisted interventions were identified, respectively. Among the highlighted facilitators, 30% of studies noted personalization and the provision of relevant tips or information, respectively. Additionally, factors such as younger age, lower severity of substance use, reinforcement, positive feedback, friendly and knowledgeable interactions, repetition of key messages, supportive relationships, immediate responses, and the perception of conversing with a human were also cited as facilitators. Conversely, reported barriers to program effectiveness included technical issues, short session durations, inappropriate responses, lack of personalization, higher severity of substance use, low readiness to change, and text-centric chatbots. However, few studies explored the statistical association between these facilitators and barriers and the program’s effectiveness. Therefore, future studies should examine this association more deeply. Nevertheless, comprehensively considering the aforementioned facilitators and barriers is crucial when developing chatbot-assisted interventions for substance use.

Recognizing the importance of chatbots resembling humans is especially crucial. This implication is evident in the use of human-like virtual agents that mimic human responses and converse with a human voice (32, 33). Regarding appearance, voice, race, and gender, the design of these chatbot avatars must avoid perpetuating biases towards specific genders, generations, races, or vulnerable populations (54). Chatbots, like humans, can acquire incorrect information or misuse it, potentially reinforcing societal biases (54).

Moreover, current chatbot-assisted programs are more useful for individuals with lower substance use severity and may be limited for those with higher levels of severity. Some studies have reported that younger users (40) and those with lower severity of substance use are more likely to actively use the applications (10, 40). Additionally, in the case of chatbot counseling, the capacity for extended, in-depth counseling and intervention is limited (19). In summary, interventions for individuals with moderate or severe substance use problems should prioritize active intervention by a professional, with chatbot-assisted programs serving as adjunctive tools until the subsequent appointment or consultation. For those with less severe substance use, chatbot programs may be more effective for prevention and early intervention. Considering this, current chatbot intervention types for prevention (7.14%) and assessment (3.57%) are very limited and need to be expanded. Furthermore, only one study (48) examined chatbot-based assessment for tobacco or alcohol use disorder. This study found the chatbot was acceptable and valid to screen for tobacco or alcohol use disorder. Therefore, developing more chatbots for prevention and assessment is necessary to enhance prevention and early intervention, particularly for young adults and youth.

Furthermore, while some studies have identified hotlines as effective responses to emergencies, including suicide (38, 42, 46), a clear protocol for detecting such crises during chatbot interactions and the post-detection process was not identified. Because substance use, such as alcohol and methamphetamine, is strongly associated with violence, suicide, and self-harm (10, 29), chatbots targeting this population must reflect intervention protocols for users in crisis.

We also suggest considering the following ethical aspects when developing chatbot-assisted programs for substance use. First, thorough security management of emotional state information, including substance use data provided by users, must be ensured. Social and moral criticism of substance use brings stigma to substance users, creating a significant barrier to their entry into treatment (5, 13). Mental health information has been cited as a sensitive area requiring special attention in AI applications (54). Thus, transparent disclosure of the retention period and disposal of such personal information may reduce user anxiety and increase trust in chatbots among substance users over the long term. Furthermore, the high usability and accessibility of chatbot services should not limit them to specific groups, such as young people and the highly educated, who are familiar with IT devices (54). Therefore, the use of these programs must be evaluated for various generations to make them accessible and comfortable for the elderly. As large-scale language models are imperfect and can be manipulated or misused based on misinformation, ongoing monitoring of the feedback and guidance provided by chatbots to users should be supervised (54) to ensure the safe delivery of interventions.

Consequently, our findings suggest that chatbot technology can facilitate ongoing interventions as an adjunctive tool without the constraints of time or place. Additionally, future research on chatbot-assisted technology for substance users requires not only more sophisticated experimental studies but also technical improvements to address ethical concerns.

This systematic review has several limitations. First, the four databases (PubMed, PsycINFO, Scopus, and CINAHL) and the keywords used to screen relevant studies may not have been exhaustive. Furthermore, because we did not conduct technical evaluations for chatbot-assisted interventions, future studies need to delve deeper into technical issues in these interventions. Additionally, due to the diverse study types (e.g., research designs) and limited number of studies with varying target variables, conducting a meta-analysis was challenging. However, as more studies accumulate, meta-analyses will become necessary. Nevertheless, our systematic review of trends in chatbot-assisted interventions (i.e., assessment, prevention, and treatment) for substance use (i.e., alcohol, smoking, and drugs) provides a valuable foundation for leveraging chatbot technology to address substance use issues. Integrating these insights into future research endeavors holds promise for advancing interventions and strategies in tackling substance use effectively.

## 5 Conclusion

This study has filled critical gaps in the literature by systematically reviewing 28 studies relevant to chatbot-assisted interventions for substance users. The results showed that the studies primarily focused on smoking and therapeutic applications, with the identified experimental studies demonstrating valid effects regardless of the theoretical approach. Chatbot programs were found to be actively used by individuals with low severity of substance use, suggesting their potential as an adjunct to interventions for substance users and as a preventive tool for adolescents and young adults. Additionally, we recommend future consideration of the ethical aspects of AI-based chatbots, particularly as they handle sensitive mental health information.

## Data Availability

The original contributions presented in this study are included in this article, further inquiries can be directed to the authors.
