# Supplementary material for: A systematic review of chatbot-assisted interventions for substance use
Source: Front Psychiatry. 2024 Sep 10;15:1456689. doi: 10.3389/fpsyt.2024.1456689 (PMC11420135; doi:10.3389/fpsyt.2024.1456689)
Supplement: Supplementary file 1 [file Table1.docx]

**Appendix 1.** Main findings

| Author  (year),  *Chatbot’s name* | Study Type | Study Design | Stage | Main Substance | Type of Intervention | Sample Size | Therapy or Theory | Duration/Session | Effectiveness- Descriptive analysis | Effectiveness- Comparative analysis | Facilitator | Barrier |
| --- | --- | --- | --- | --- | --- | --- | --- | --- | --- | --- | --- | --- |
| Abdullah et al. (2018), *ECA-Q* [44] | Quan | Non-experimental | Test | Smoking | Treatment | 6 | US Clinical Practice Guidelines | 2 weeks/  14 sessions | - 100% (Helpful for smoking)  - 50% (Setting a quit smoking date within 14 days)  - 83.33% (Reduced smoking)  - 100% (Positive feelings, "impressive and satisfaction")  - 83.3% (Easy interaction)  - 66.67% (Felt it was lifelike) | N/A | - Providing relevant tips or information  -Reinforcement and positive feedback  - Friendly and knowledgeable interactions  - Repetition of key messages  - Supportive interpersonal relationships | - Technical problems  - Short session durations  -Inappropriate responses |
| Albers et al. (2022), *Sam* [29] | Mixed | Non-experimental | Test | Smoking | Treatment | N/A | N/A | 5 sessions | N/A | N/A | N/A | N/A |
| Almusharraf et al.  (2020),  *Motivational Interviewing-based Chatbot* [53] | Quan | Non-experimental | Test | Cigarette | Treatment | 121 | N/A | 14 weeks | - 8.3% (Helpful for smoking)  - 34.7% (Positive feelings, "pleasant") | N/A | N/A | N/A |
| Alphonse et al. (2022),  *Quit Coach* [36] | Qual | Non-experimental | Test | Smoking | Treatment | 14 | N/A | 2 weeks | - 33.33% (Quit smoking)  - 66.67% (Cut down smoking) | N/A | N/A | N/A |
| Auriacombe et al. (2018),  *ECA Jeanne* [48] | Quan | Quasi-experimental | Test | Tobacco  and Alcohol | Assessment | 139 | N/A | N/A | - High acceptability E-Scale scores (24.8; out of 30, *SD* = 4.2) | - High scores of correlation between the ECA, CDS-5, and CAGE interviews and the paper version questionnaires (*r*(139) = .944, *p* < .0001 for CDS-5 and *r*(139) = .893, *p* < .0001 for CAGE) | N/A | N/A |
| Avila-Tomas et al. (2019),  *Dejal@bot* [37] | Quan | Experimental | Plan | Smoking | Treatment | 460 | Convergence (Gamification, CBT, MI, problem-solving, and relapse-preventing components) | N/A | N/A | N/A | N/A | N/A |
| Barnett et al. (2021),  *N/A* [18] | Qual | Non-experimental | Plan | Substance | N/A | 28 | N/A | N/A | N/A | N/A | N/A | N/A |
| Bendotti et al. (2024),  *Quin* [33] | Mixed | Non-experimental | Plan | Smoking | Treatment | N/A | N/A | N/A | N/A | N/A | -Personalization | N/A |
| Boustani et al. (2021),  *Digital Health Agent* [32] | Mixed | Non-experimental | Test | Alcohol | Treatment | 51 | Brief MI | 1 week | - High acceptability and utility of the technology (*M* = 2.31, *SD* = 1.05, out of 7)  - High engagement (*M* = 2.86, *SD* = 0.96, out of 7)  - High number of human-like traits (*M* = 2.07, *SD* = 0.89, out of 7) | N/A | N/A | N/A |
| Brown et al. (2023),  *MIBot* [40] | Quan | Quasi-experimental | Test | Smoking | Treatment | 349 | MI | 1 session | - 25%-40% (Attempt to quit)  - 70.5% (Make some kind of smoking reduction attempt) | - Quitting confidence (*M* change 0.1, *SD* = 2.0-2.3, *p* < .001)  - Quitting importance (*M* change = 0.7, *SD* = 2.0, *p* < .001)  - Quitting readiness (*M* change 0.4, *SD* = 1.7, *p* < .01) | - Younger age  - Lower severity of substance use | -Inappropriate responses |
| Chen et al. (2023),  *Echo-App* [50] | Quan | Quasi-experimental | Test | Methamphetamine | Treatment | 47 | N/A | 1 session | N/A | - Increased treatment motivation for substance use (*p* < .001, *Cohen’s d* = -0.60)  - Increased motivation for abstaining from drugs (*p* = .045, *Cohen’s d* = -0.30)  - Increased confidence for substance or drug use (*p* < .01, *Cohen’s d* = -0.45)  -Increased importance for substance or drug use (*p* < .001, *Cohen’s d* = -0.50)  - Decreased craving (*p* = .01*, Cohen’s d* = .038) | - Patients’ scores on the Generalized Anxiety Disorder-7 assessment, Barratt Impulsiveness Scale-Motor Impulsiveness | N/A |
| Chun-Hung et al. (2023),  *CAT* [9] | Quan | Experimental | Test | Methamphetamine | Treatment | 99 | MBRP | 6 months | - 85% (Helpful for methamphetamine use disorder)  - 67% (Positive feelings, "cared"), 84% (Positive feelings “satisfaction”)  - 67% (Would recommend) | - Fewer methamphetamine-positive urine samples (19.5% in experimental group vs 29.6% in control group, *F* = 9.116, *p* = .003) | - Immediate access to responses | - Higher severity of substance use  - Low readiness to change |
| Dulin et al. (2022),  *Step Away* [47] | Quan | Experimental | Test | Alcohol | Treatment | 150 | Convergence (Person-centered, behavioral-based intervention and the treatment of addictions: motivational enhancement, relapse prevention, community reinforcement) | 12 weeks | - Predict reduced drinking (β = 0.25, 95% *CI* = 0.00-0.01, *p* = .04) | N/A | - Perception of conversing with a human | - Technical problems  - Text-centric chatbots |
| Emezue et al. (2023),  *BrotherlyACT* [28] | Mixed | Quasi-experimental | Plan | Substance | Treatment | N/A | Acceptance and Commitment Therapy | 10 weeks | N/A | N/A | N/A | N/A |
| Haug et al. (2023)  , *Mobile Coach Alcohol* [52] | Quan | Quasi-experimental | Test | Alcohol | Prevention | 954 | Convergence (CBT, social norms approach, MI) | 10 weeks | - 84.6% (Helpful for alcohol)  - 87.9% (Positive feelings, "rate enjoyed")  - 76.2% (Would recommend)  - 89.1% (Would participate again)  - 100% (Comprehensibility)  - 70.8% (Relevant to the individual situation) | - Decreased binge drinking (*OR* = 0.32, 95% *CI* = 0.18-0.57, *p* < .001)  - Decreased maximum alcohol consumption (*IRR* = 0.75, 95% *CI* = 0.68-0.82, *p* < .01)  - Decreased number of standard drinks per month (*IRR* = 0.62, 95% *CI* = 0.58-0.67, *p* < .01)  - Increased drinking refusal self-efficacy (*β* = 0.24, 95% *CI* = 0.06-0.42, *p* = .01) | N/A | N/A |
| Haug et al. (2022),  *Ready4life* [43] | Quan | Experimental | Test | Alcohol  and Smoking | Prevention | 1,351 | Convergence (CBT, social norms approach, MI) | 8 weeks | N/A | - Significant group effects for the 30 days point prevalence for tobacco/e-cigarette smoking (${OR}_{ITT}$ = 0.74, 95% *CI* = -0.55-1.01, ${OR}_{CC}$ = 0.62, 95% *CI* = 0.40-0.96)  - Significant group effects for at-risk drinking in the past 30 days (${OR}_{ITT}$ = 0.68, 95% *CI* = 0.52- 0.89, ${OR}_{CC}$ = 0.61, 95% *CI* = 0.43-0.84)  - Significant group effects for total number of alcoholic drinks consumed in the past 30 days (*Cohen’s d_ITT_* = 0.07, *Cohen’s d_cc_* = 0.11)  - Decreased number of cannabis use days in past month (*Cohen’s d_ITT_* = 0.06, *Cohen’s d_cc_* = 0.14) | -Personalization | N/A |
| He et al. (2022), *Roby* [41] | Quan | Experimental | Test | Smoking | Treatment | 153 | MI | 2 sessions | N/A | - Motivation to quit (*F* (1,151) = 32.67,  *p* <.001) | - Providing relevant tips or information | - Short session durations |
| Leeuwis & He. (2022),  *Cecil* [49] | Quan | Experimental | Test | Smoking | N/A | 233 | MI | 1 session | N/A | - Smoker’s intention to quit (*M* change 0.8, *SE* = 0.1,  *p* < .001, respectively) | N/A | N/A |
| Loveys et al. (2023), *Florence* [31] | Mixed | Non-experimental | Test | Tobacco | Treatment | 115 | N/A | N/A | - Positive overall experience (*M* = 3.17, *SD* = 0.82, out of 4)  - Chatbot provide useful information and advice (*M* = 3.21, *SD* = 0.92, out of 4). |  | N/A | N/A |
| Monteiro et al. (2022),  *Pahola* [34] | Mixed | Non-experimental | Test | Alcohol | Treatment | N/A | N/A | N/A | - 50% (Choosing to cut back on drinking) |  | N/A | N/A |
| Nair et al. (2023),  *N/A* [30] | Mixed | Quasi-experimental | Plan | Tobacco | Treatment | 300 | N/A | 2 weeks | N/A |  | N/A | N/A |
| Olano-Espinosa et al. (2022), *Dejal@bot* [51] | Quan | Experimental | Test | Smoking | Treatment | 513 | Convergence (Gamification, CBT, MI, problem-solving, and relapse-preventing components) |  | N/A | - Biochemically validated abstinence rate of smoking (26% for intervention group vs 18.8% in control group, *OR* = 1.52, 95% *CI* = 1.00-2.31, *p* = .05) | N/A | N/A |
| Perski et al. (2019),  *Smoke Free app* [45] | Quan | Experimental | Test | Smoking | Treatment | 57,214 | N/A |  | N/A | - Quit success (79.55% in intervention group vs 73.35% in control group, ${OR}_{adj}$ = 1.36, 95% *CI* = 1.16-1.61, *p* < .001) | N/A | N/A |
| Prochaska et al. (2021a),  *Woebot-SUDs* [42] | Quan | Experimental | Test | Substance | Treatment | 180 | Convergence (CBT, DBT, MI, mindfulness training) | 8 weeks | - 96% (Positive feelings, "rate positively")  - 86% (Would recommend) | - Decreased past-month substance use occasions (*M* change -9.1, *SE* = 2.0 in intervention group vs *M* change = -3.3, *SE* = 1.8 in control group; *p* = .039, ${Eta}^{2}$ = .029) | N/A | N/A |
| Prochaska et al. (2023),  *Woebot-SUDs* [38] | Quan | Quasi-experimental | Plan | Substance | Treatment | 400 | Convergence (CBT, DBT, MI) | 8 weeks | N/A |  | N/A | N/A |
| Prochaska et al. (2021b),  *Woebot-SUDs* [46] | Quan | Quasi-experimental | Test | Substance | Treatment | 101 | Convergence (CBT, DBT, MI, mindfulness training) | 8 weeks | - 94% (Positive feelings, "rate positively") | - Decreased scores on the AUDIT-C (*M* change -1.3, *SD* = 2.6, *p* < .001)  - Increased confidence for substance or drug use (*p* < .01, Cohen’s d = -0.45)  - Decreased craving (*M* change -0.38, *B*(*SE*) = −0.38(0.16), *OR* = 0.69, 95% *CI* = 0.50-0.90)  - Decreased past-month substance use occasions (*M* change -9.3, *SD* = 14.1, *p* < .001)  - Decreased scores on the DAST-10 (*M* change -1.2, *SD* = 2.0, *p* < .001) | N/A | N/A |
| Sedotto et al. (2024),  *Step Away chatbot* [35] | Qual | Non-experimental | Test | Alcohol | Treatment | 20 | N/A | 12 weeks | N/A | N/A | -Personalization  - Providing relevant tips or information | -Inappropriate response  - Lack of personalization |
| Singh et al. (2024),  *N/A* [39] | Mixed | Non-experimental | Plan | Nicotine | Treatment | NA | Behavioral theory | N/A | N/A | N/A | N/A | N/A |

Note *N/A, Non-Applicable; Mixed, Mixed methods research; Qual, Qualitative research; Quan, Quantitative research.*
